# Tubercle Infection

**Published:** 1889-12-07

**Authors:** 


### TUBERCLE INFECTION.

Dr. Cornet, of Berlin, has lately insisted on the fact, observed by others, that the breath of tuberculous persons does not contain any bacilli, but that they are confined to the sputa, and that, their vitality not being destroyed by dessication, infection takes place only when they are diffused as dust through the air from sputa allowed to dry on the floor, or in handkerchiefs. Dr. Beaumetz reports a striking illustration of this danger. In a large establishment in Paris, where twenty-two clerks were employed for eight hours daily in an ill-ventilated room, giving only 300 cubic feet per head, no fewer than fourteen have died of tuberculosis since the first, eleven years ago. They had been in the habit of expectorating on the floor, which was ill laid and rough, and swept each morning, the men often entering while the air of the office was charged with the dust raised by the process.

